# Emotion Regulation in Binge Eating Disorder: A Review

**DOI:** 10.3390/nu9111274

**Published:** 2017-11-22

**Authors:** Alexandra Dingemans, Unna Danner, Melissa Parks

**Affiliations:** 1 Rivierduinen Eating Disorders Ursula, 2300 AK Leiden, The Netherlands; m.parks@rivierduinen.nl; 2 Institute of Psychology, Leiden University, 2333 AK Leiden, The Netherlands; 3 Altrecht Eating Disorders Rintveld, 3705 WE Zeist, The Netherlands; u.danner@altrecht.nl; 4 Department of Clinical Psychology, Utrecht University, 3584 CS Utrecht, The Netherlands

**Keywords:** Binge Eating Disorder, review, emotion, regulation, negative mood, anger, suppression

## Abstract

The purpose of the present review is to provide a summary of the research findings on emotion regulation in Binge Eating Disorder (BED). Negative emotions and maladaptive emotion regulation strategies play a role in the onset and maintenance of binge eating in BED. Anger and sadness, along with negative emotions related to interpersonal experiences (i.e., disappointment, being hurt or loneliness), seem to be particularly relevant. Individuals with BED have a tendency to suppress and ruminate on their unwanted emotions, which leads to increased psychopathological thoughts and symptoms. Compared to healthy controls, they use adaptive strategies, such as reappraisal, less frequently. Evidence concerning the causal relation between negative affect and binge eating is inconclusive and still very limited. While experimental studies in a laboratory setting lack ecological validity, ecological momentary assessment studies offer more promise at unraveling the causal relationship between emotions and binge eating. Increases in negative affect are found to be antecedents of binge eating in BED. However, there seems to be less support for the possibility that binge eating serves as a means to alleviate negative affect. Finally, BED seems to be related to other forms of maladaptive emotion regulation strategies, such as substance abuse and self-harm.

## 1. Introduction

One of the key symptoms of Binge Eating Disorder (BED) is eating large amounts of food within a limited period of time while experiencing feelings of loss of control [1]. Many sufferers eat alone due to shame and are tormented by feelings of disgust, guilt or sadness afterwards [2]. The most recent Diagnostic and Statistical Manual of Mental Disorders DSM-5 criteria [1] for this illness, acknowledge that binge eating and negative emotions are linked, requiring the presence of “marked distress regarding binge eating” in order to receive a diagnosis of BED. Previous systematic reviews and meta-analyses on emotion regulation in BED have limited the scope of their results to findings from experimental studies [3,4,5] or to studies with control groups [6], or they have focused on obesity [7] rather than specifically BED. The present review aims to provide a broader overview of the research findings on emotion regulation in BED.

First, an overview of the findings regarding emotions in individuals with BED will be presented. Next, findings concerning the use of adaptive and maladaptive emotion regulation strategies among individuals with BED will be presented. This will be followed by an explanation of the theoretical models concerning the causal relationship between emotions and binge eating, as well as a summary of the evidence from experimental and ecological momentary assessment (EMA) studies regarding this relationship. Following this, findings regarding the causal relationship of emotional regulation strategies will be presented. Finally, the concept of negative urgency, and the role it may play in the relationship between emotion regulation and binge eating, will be briefly examined.

A literature review was conducted in the following electronic databases: Pubmed, PsychINFO and Embase. Studies were selected if they met the following criteria: (1) Language in English (2) published in peer reviewed journals and (3) available abstract. The following search terms were applied: (binge eating disorder or “binge eating”) and (emotion or affect, or mood or stress) combined with (regulation). The reference lists of retrieved articles were also checked for other relevant studies.

## 2. Emotions in Individuals with BED

Several decades ago, Bruch [8] suggested that there might be a link between emotional factors and overeating. More recent research findings reveal that the majority of individuals with BED present at least one lifetime comorbid psychiatric disorder (67% to 79%), with mood and anxiety disorders amongst the most prevalent [9,10,11]. In addition to mood being overall worse among individuals with BED, it is especially poor directly prior to binge eating. Greeno et al. [12] investigated binge antecedents in women with BED and concluded that poor mood directly preceded binge episodes. Depressive mood (i.e., sadness) has been the most frequently examined negative emotion in this disorder [13]. Several cross-sectional, experimental and therapy outcome studies suggest an association between depressive symptoms, acute sad mood, and binge-eating behavior, and indicate that higher levels of depression are related to more severe binge eating [14,15,16,17,18,19,20]. In addition, food cravings that ended in a binge were associated with lower levels of mood, energy and hunger, and higher levels of tension, than cravings that did not lead to a binge [21]. Finally, negative mood ratings in BED appear to be higher, whereas positive mood ratings are lower, on binge days compared to non-binge days [22,23].

In addition to sadness, other emotions have also been found to play a role in binge eating. While anxiety appears to be of less importance than other emotions in the context of binge eating [24,25,26], Arnow et al. [25] found that anger/frustration, anxiety and sadness/depression accounted for 95% of the moods preceding a binge-eating episode. Anger and frustration in particular, preceded the urge to binge more often than sadness and depression. This dimension, ‘anger/frustration’, included negative emotions involving others (e.g., discouraged, guilty, irritated, angry, furious, inadequate, helpless, resentful, frustrated, jealous, rebellious) [25], which all arise from an interpersonal context [27]. Anger in particular seems to be an especially important emotion in binge eating [24,27,28,29]. Investigating a broad spectrum of emotions in BED, Zeeck et al. [24] found that the number of binges was best explained by anger, disappointment and feelings of being hurt or lonely. These authors also concluded that emotions that are related to interpersonal experiences seem to be particularly relevant in BED [24]. Other studies have suggested that interpersonal problems lead to greater negative affect, which results in higher levels of binge eating in BED [30,31,32,33].

It also appears that individuals with BED experience stressors and the resulting emotions differently than their healthy peers. For example, research on stressors and emotional state in individuals with BED found that they reported experiencing more negative stressors (in the areas of work/school, family, friends, the environment, and practical considerations), and were less able to tolerate negative mood, when compared to healthy controls [34,35]. In addition, daily hassles were experienced as more stressful by individuals with BED than a control group with a similar number of reported daily hassles [34]. Finally, according to a systematic review on the emotional functioning of individuals with BED, they present greater difficulties in terms of emotional awareness (i.e., alexithymia, interoceptive awareness, and clarity) as compared to normal-weight and obese individuals without an eating disorder diagnosis [6].

While some studies have explored the relationship between positive affect and eating in healthy populations (e.g., [4,36,37,38]), very few have been carried out exploring the relationship between eating and positive emotions specifically in BED [23,24,39]. De Young et al. [40] found that on weeks with lower-than-usual negative affect and higher-than-usual positive affect, individuals with binge-eating psychopathology (bulimia nervosa (BN) and BED) reported fewer binge-eating episodes, although the effect size was relatively small. Overall, knowledge regarding associations between positive emotions and eating in BED is very limited and additional studies are needed [13].

## 3. Emotion Regulation Strategies in BED

Emotion regulation is a term generally used to describe a person’s ability to effectively manage and respond to everyday events [41] and refers to the processes by which we influence which emotions we have, when we have them, and how we experience and express them [42]. People unconsciously use emotion regulation strategies to cope with difficult situations many times throughout the day. Individuals who regulate their emotions in an adaptive way have the abilities to experience and differentiate, as well as attenuate and modulate, these affective states [43]. Many models suggest that individuals engage in binge eating as a means to down-regulate negative emotions, which is likely due to lacking more adaptive strategies [41,44]. The existing literature suggests that binge eating may temporarily improve one’s mood (e.g., [39,45,46]. In other words, the problem is not necessarily associated with the experience of negative emotions per se, but rather with their lack of adaptive strategies to regulate negative affect [47].

Although the number of emotion regulation strategies is potentially limitless [42], examples of both adaptive and maladaptive strategies include reappraisal, problem solving, acceptance, avoidance, rumination and suppression [41]). Reappraisal is a prototypical adaptive emotion regulation strategy [48], which involves generating benign or positive interpretations or perspectives on a stressful situation, as a way to decrease its emotional impact [41]. This strategy is an example of an antecedent-focused strategy, which means that an individual uses this strategy before the emotion response tendencies have become fully activated and have influenced their behavior [42]. Another adaptive strategy is problem solving, which involves making conscious attempts to change a stressful situation, or to contain its consequences. Although problem-solving responses are not direct attempts to reduce emotional distress, they tend to have beneficial effects at modifying or eliminating stressors [41].

One example of a maladaptive emotion regulation strategy is that of suppression, which is considered to be a response-focused strategy [42], and is commonly used by people with eating disorders [41]. When an individual uses suppression as a regulation strategy, it is the expression of the emotions, rather than the experience of the emotions, that is reduced. This strategy may be effective in the short term, however, is likely to fail in the long run [42]. Eventually this strategy leads to greater physiological arousal, and is not effective in reducing emotions. Several studies showed that people often fail at trying to suppress a negative emotion [49,50,51]. Eventually, suppression seems to have the opposite effect from that which is desired and leads the person to fixate on the emotion. Chronic suppression might prevent someone from becoming more accustomed to emotional stimuli, thus leading to more psychopathological thoughts and symptoms [51]. A second maladaptive regulation strategy [41] that has been associated with BED is rumination. People with BED engage in rumination in an attempt to understand and solve their problems, despite the fact that it tends to interfere with effective problem solving and may lead to indecision [41].

Studies investigating the use of emotion regulation strategies amongst individuals with BED have found mixed results. When compared to healthy controls, individuals with BED have a tendency to suppress their emotions more and reappraise their emotions less [52]. Several studies found a positive relationship between anger suppression and bulimic tendencies [53,54]. When the differences with regards to the type of emotion regulation strategies that were used between the eating disorder subtypes (anorexia nervosa (AN), BN and BED) was examined, Svaldi et al. [55] found no major differences. However, Danner et al. [48] showed that individuals with BED (similar to BN and AN binge/purge subtype) seemed to be less inclined to reappraise their emotions than women with AN, restrictive subtype. Moreover, they seemed to use this adaptive strategy even less when the state of their illness was worse. Moreover, individuals with BED provided a worse evaluation of their ability to influence their emotional states than those individuals with other eating disorder subtypes (AN, BN and eating disorder not otherwise specified) [56]. However, other studies [43,52], reported less emotion regulation difficulties in BED than in the other eating disorder subtypes.

To our knowledge, no studies have compared BED with other groups with respect to the use of acceptance and rumination, although rumination has been associated more generally with bulimic behaviors [41,57]. In individuals with BED, rumination, especially that which involves a passive comparison of one’s current situation to desired standards (i.e., brooding rumination), appears to lead to more negative mood [58,59]. Wang et al. [58] suggested that rumination is an important cognitive process associated with the severity of eating disorder psychopathology in BED. That is, those who dwell on their current body size and compare it with desired standards may be especially concerned and distressed about their body shape.

The maladaptive emotion regulation strategies of individuals with BED may affect not only eating pathology, but other behaviors as well. For example, substance abuse [9,10,11] and impulse control problems [9] are often co-morbid to BED. BED and substance use disorders share a number of psychological, neurobiological and genetic correlates [60], and mood dysregulation is associated with both disorders. Also, self-injury is frequently associated with impulsivity and substance use [61] and appears to be present in individuals with BED as well, to the same the degree as the other eating disorder subtypes (>20%) [62]. In conclusion, it appears that difficulties with emotion regulation are an important factor involved in the maintenance of eating disorder psychopathology, and may manifest in other forms of psychopathology as well. While individuals with BED appear to use more maladaptive emotion regulation strategies than healthy controls, only subtle differences exist between the eating disorder subtypes [55]. Additional studies are needed in order to continue to explore the use of these strategies specifically in individuals with BED.

## 4. Empirical Evidence for the Theoretical Models of BED

Most theoretical models of eating disorders [63,64,65] incorporate the theory that emotions are often poorly regulated and that sufferers often turn to food to escape from, or down-regulate, their emotions [41]. For example, in their transdiagnostic model, Fairburn et al. [63] suggest that mood intolerance, referred to as an inability to appropriately cope with emotional states, may be an underlying process that is involved in the maintenance of all eating disorders. This intolerance may apply to all intense mood states, including positive ones (e.g., excitement) and the more common aversive negative ones (e.g., depression, anger, anxiety) [63]. In individuals with eating disorders, binge eating most commonly serves as an attempt to regulate these undesired mood states. A model that focuses more specifically on binge eating is the affect regulation model [66]. This model posits that increases in negative emotions trigger binge-eating episodes, and that binge eating serves as a means to alleviate negative affect by using food for comfort and distraction. According to the escape from self-awareness model, binge eating is used as a means to escape from negative mood and to help alleviate emotional stress [65]. According to this model, binge-eating episodes can be seen as the result of an individual’s effort to draw attention away from emotional distress and an aversive self-perception, and towards a narrower focus on the immediate environment (i.e., food).

Leehr et al. [3] suggested that these models and theories have two components in common: (1) Negative emotions serve as a trigger for binge eating and (2) binge eating serves as a means to down-regulate (i.e., relief component) these negative emotions in the short-term (during the binge eating episode) or in the longer term (after the binge-eating episode). These causal relationships have been investigated by means of both experimental studies and EMA studies.

Experimental studies have a few elements in common. First, participants need to be randomly assigned to two or more (negative, positive and/or neutral) emotion induction conditions. Second, in order to allow for a good comparison between the conditions, mood has to be measured before and after the mood induction, and the mood induction has to be successful (i.e., significant changes in mood as a result of the induction). Finally, participants are asked to complete either a bogus taste task with caloric intake calculated afterward, or a questionnaire that measures the desire to eat. Several experimental studies have investigated the causal relationship between emotions and eating behavior in BED [39,67,68,69,70,71,72,73,74,75,76]. Some of these studies [72,74,77,78] found that compared to the (obese) control groups, individuals with BED ate more or had a greater desire to eat after a negative mood induction. Other studies revealed that participants with BED that underwent negative mood induction demonstrated greater caloric intake or urge to eat than participants with BED in the control condition [67,68]. However, other studies did not find such a relationship [39,46,69]. Overall, the number of experimental studies is very limited in BED and the results are mixed, which is mirrored in the conclusions of recent systematic reviews [3] and meta-analyses [4,5,79]. For example, in their systematic review, Leehr et al. [3] concluded that the trigger component proposed by the escape theory [65] is potentially true. Cardi et al. [4] found in their meta-analysis that negative mood was associated with greater food intake in samples of binge eaters. However, in a recent meta-analysis (in preparation) Evers et al. [79] concluded that negative and positive emotions were not associated with changes in eating behavior in BED in laboratory settings, and thus, no causal effect was found of emotion on eating behavior.

One of the disadvantages of experimental studies is the limited ecological validity of the findings outside the laboratory setting as these settings are often very different from one’s natural environment. A promising research design which may overcome some of these problems is EMA [5]. Studies employing EMA typically ask individuals to complete several ratings concerning their daily experiences, behavior, and psychological states at fixed or random times during the day, while in their natural environment. EMA aims to minimize recall bias, maximize ecological validity, and allows participants to report on symptoms, affect, behavior and cognitions close in time to experiences and in real-world contexts [80]. EMA studies have demonstrated that binge eating in BED is preceded by the experience of negative emotions [22,23,81,82,83], which provides further evidence for the premise that high or increasing negative affect serves as an antecedent, or trigger, for binge eating. A meta-analysis of studies using EMA concluded that increases in negative affect were antecedents of binge eating in both BED and BN [5], but that individuals with BED experienced larger increases of negative affect prior to binge eating compared to individuals with BN. This meta-analysis was not able to find support for the second key prediction of the affect regulation model (i.e., that binge eating serves as a means to alleviate negative affect) as findings suggested that negative affect actually increased after binge-eating episodes in both BN and BED. However, a more recent EMA study that was not included in this meta-analysis did find that binge eating decreased negative affect [83]. Additional EMA studies assessing the relationship between binge-eating episodes and subsequent changes in affect are needed in order to increase our understanding of the effect that binge eating may have on mood in naturalistic settings.

## 5. The Causal Effect of Emotion Regulation Strategies on Binge Eating

As far as we know, only a few studies have investigated the causal relationship between emotion regulation strategies and overeating in BED [19,52,71]. Svaldi et al. [52] instructed 27 women with BED and 25 healthy control women to do the following while watching sadness-inducing film clips: (1) Watch the clips; (2) watch the clips and suppress or; (3) watch the clips and reappraise the emotions that arose. Following this, the desire to binge was measured by means of a questionnaire. They found that suppression of negative emotions, but not reappraisal, led to a greater desire to binge in women with BED when compared to healthy control women. Two other studies [19,71] instructed individuals to regulate their emotions during negative mood induction, which was followed by a bogus taste task, with mixed results. In the study by Dingemans et al. [19], individuals with BED were instructed to either act natural during a negative mood induction or to suppress their emotions while watching. No differences were found between the two conditions in regards to caloric intake during the taste task and thus, the hypothesis that suppression leads to overeating in BED was not supported. However, in the experiment by Svaldi et al. [71], women with BED and a weight-matched control group were first provided with training in emotional regulation by means of suppression or cognitive reappraisal. Subsequently, they were randomized to a negative mood induction were they were instructed to use one of the two emotion regulation strategies followed by a taste task. Both groups consumed more calories in the suppression condition than in the reappraisal condition. Overall, the BED group consumed more calories than the weight-matched control group. Differences in the results of these studies may be explained by the fact that individuals with BED tend to suppress their emotions more often and reappraise them less often. Thus, it is possible that participants in the control condition (‘act natural’) still engaged in suppression (although to a lesser extent) since this is their natural tendency [71]. Additional experimental studies are needed to further explore the effect of specific emotion regulation strategies on binge eating.

## 6. The Role of Negative Urgency in BED

Impulsivity is a concept that has often been linked to binge-eating pathology in BN and BED [84] and may play a role in the relationship between negative affect and binge eating. A meta-analysis of studies on impulsivity in relation to bulimic symptoms [85] concluded that negative urgency (i.e., emotion-based dispositions to act rashly) appeared to be particularly relevant and was much more strongly linked to bulimic symptomatology than other facets of impulsivity, such as motor inhibition or non-planning. Since then, several studies have replicated these findings (e.g., [86,87]), although the majority of studies have included individuals displaying binge-eating pathology without distinguishing between eating disorder diagnoses (e.g., [88,89]). Wolz et al. [90] confirmed that individuals with BN, BED and other unspecified feeding and eating disorders presented similar levels of negative urgency. In a recent small study, negative urgency predicted binge eating frequency and global eating pathology during and after treatment [91], with a higher level of negative urgency predicting a smaller reduction of binge-eating frequency and global eating pathology. Negative urgency has also been examined in an experimental study. Danner et al. [92] demonstrated that when negative affect was induced, women with BN and BED behaved more impulsively (i.e., they chose more disadvantageously on a decision-making task) after receiving a punishment, whereas healthy control women did so after receiving a reward. It may be that further increases in negative feelings caused by the punishment (and in contrast to the reward) may have triggered the impulsive behavior, thus providing further support for the presence of negative urgency in BN and BED. Future research would benefit from using experimental designs to test whether maladaptive emotion regulation moderates or mediates the link between negative urgency and binge-eating pathology as a means to gain more insight into the role of negative urgency on the pathological behaviors of individuals with BED.

## 7. Conclusions

The scientific literature provides clear evidence that negative emotions and maladaptive emotion regulation strategies play a role in the onset and maintenance of binge eating in BED. Poor mood appears to precede binge-eating episodes and as is proposed by the theoretical models of BED, binge eating may be an attempt to down-regulate this emotional distress. This may be related to the fact that individuals with BED appear to lack healthy and effective emotion regulation strategies and have a tendency to suppress their unwanted emotions, engage in rumination and use adaptive strategies, such as reappraisal, far less than healthy controls. Furthermore, their attempts to down-regulate their unwanted emotions may also lead to other unhealthy behaviors such as substance abuse and self-injury. Additional studies are needed to further unravel the link between emotions, emotion regulation and (pathological) behaviors presented by individuals with BED. Future research may benefit from utilizing EMA to assess the relationship between emotional distress and binge eating as a means to provide more empirical support for the theoretical models explaining BED. Furthermore, additional studies are needed in order to clarify the differences in the role played by emotion dysregulation between BED and BN. Whereas BN binge-cycles normally end with purging behavior and thus immediate, albeit short-term, relief, binge eating in BED may result in an increase in negative affect [23]. Another direction of future research includes exploring the role of positive emotions in BED and their relationship with binge-eating episodes. Finally, future studies could further investigate the effect that specific emotion regulation strategies have on binge eating. Together, these findings could provide valuable insights into the emotional mechanisms underlying BED and possible avenues for improving treatment interventions.
